# Biomedical literature-based clinical phenotype definition discovery using large language models

**DOI:** 10.1093/database/baaf047

**Published:** 2025-09-24

**Authors:** Samar Binkheder, Xiaofu Liu, Michael Wu, Lei Wang, Aditi Shendre, Sara K Quinney, Wei-Qi Wei, Lang Li

**Affiliations:** Medical Informatics Unit, Department of Medical Education, College of Medicine, King Saud University, Riyadh 12372, Saudi Arabia; Department of Biomedical Informatics,, College of Medicine, Ohio State University, 1800 Cannon Drive, Columbus, OH 43210, United States; Department of Biomedical Informatics,, College of Medicine, Ohio State University, 1800 Cannon Drive, Columbus, OH 43210, United States; gRED Computational Sciences, Computational Biology & Translation, Genentech, Inc, 1 DNA Way, South San Francisco, CA 94080, United States; Department of Biomedical Informatics,, College of Medicine, Ohio State University, 1800 Cannon Drive, Columbus, OH 43210, United States; Department of Biomedical Informatics,, College of Medicine, Ohio State University, 1800 Cannon Drive, Columbus, OH 43210, United States; Department of Obstetrics and Gynecology, School of Medicine, Indiana University, 950 W Walnut Street, Indianapolis, IN 46202, United States; Department of Biomedical Informatics, Vanderbilt University Medical Center, 2525 West End Ave, Nashville, TN 37203, United States; Department of Biomedical Informatics,, College of Medicine, Ohio State University, 1800 Cannon Drive, Columbus, OH 43210, United States

## Abstract

Electronic health record (EHR) phenotyping is a high-demand task because most phenotypes are not usually readily defined. The objective of this study is to develop an effective text-mining approach that automatically extracts clinical phenotype definitions-related sentences from biomedical literature. Abstract-level and full-text sentence-level classifiers were developed for clinical phenotype discovery from PubMed. We compared the performance of the abstract-level classifier on machine learning algorithms: support vector machine (SVM), logistic regression (LR), naïve Bayes, and decision tree. SVM classifier showed the best performance (F-measure = 98%) in identifying clinical phenotype-relevant abstracts. It predicted 459 406 clinical phenotype-related abstracts. For the full-text sentence-level classifier, we compared the performance of SVM, LR, naïve Bayes, decision trees, convolutional neural networks, Bidirectional Encoder Representations from Transformers (BERT), and Bidirectional Encoder Representations from Transformers for Biomedical Text Mining (BioBERT). BioBERT model was the best performer among the full-text sentence-level classifiers (F-measure = 91%). We used these two optimal classifiers for large-scale screening of the PubMed database, starting with abstract retrieval and followed by predicting clinical phenotype-related sentences from full texts. The large-scale screening predicted over two million clinical phenotype-related sentences. Lastly, we developed a knowledgebase using positively predicted sentences, allowing users to query clinical phenotype-related sentences with a phenotype term of interest. The Clinical Phenotype Knowledgebase (CliPheKB) enables users to search for clinical phenotype terms and retrieve sentences related to a specific clinical phenotype of interest (https://cliphekb.shinyapps.io/phenotype-main/). Building upon prior methods, we developed a text mining pipeline to automatically extract clinical phenotype definition-related sentences from the literature. This high-throughput phenotyping approach is generalizable and scalable, and it is complementary to existing EHR phenotyping methods.

## Introduction

Phenotype definitions are crucial for identifying patients with a specific disease or condition in electronic health records (EHRs). Within an EHR context, ‘a phenotype is defined as a biochemical or physical trait of an organism, such as a disease, clinical or physical characteristics, or blood type’ [1]. These phenotypes can be queried from EHR repositories using different methods, including defined data element sets and logical expressions [2]. EHR-phenotyping is a broad concept defined by the National Institutes of Health (NIH) Health Care Systems Collaboratory as ‘activities and applications that use EHR data exclusively to describe clinical characteristics, events, and service patterns for specific patient populations’ [2, 3]. Phenotyping applications in EHR-based studies include cross-sectional (e.g. epidemiological research), case-control/cohort associations (e.g. Pharmacovigilance), and experimental (e.g. clinical trial recruitment) [4], which can offer several advantages, including cost-effectiveness and real-world clinical data. Unfortunately, a primary informatics challenge of EHR phenotyping is the precise phenotype definition, especially when phenotype definitions are not readily available [5, 6]. The need to assess and improve data capture for defining study populations and outcomes was one of the EHR-related challenges identified by the NIH Health Care Systems Research Collaboratory initiative that aims to facilitate large-scale pragmatic clinical trials (PCTs) nationally in the United States [7].

Developing EHR phenotypes is an intricate and labour-intensive process that demands extensive expertise in both the clinical and informatics domains [8–10]. Phenotyping algorithms typically fall into three categories: rule-based, machine-learning-based, or high-throughput-based. Rule-based algorithms rely on clinical experts to select specific criteria (e.g. diagnosis codes, medications, and laboratory values) to define a phenotype, such as type 2 diabetes mellitus (T2DM) [11] and major adverse cardiac events (MACE) on Statin [12, 13]. Kho et al. [11] developed a T2DM rule-based algorithm where the process started with the clinical diagnostic criteria established by the American Diabetes Association to identify T2DM cases and controls based on diagnostic codes, medications, and laboratory tests (e.g. glucose levels). Their algorithm demonstrated high positive predictive values of 98% for cases and 100% for controls across five institutions. However, rule-based phenotype may miss some cases, i.e. suboptimal recall.

Machine learning (ML)-based approaches commonly use data from a single institution and can capture nuanced phenotypes such as visit frequency. They can broadly be categorized into supervised and unsupervised methods. As an example of supervised ML-based method, Zhao et al. [14] conducted a comprehensive analysis using various ML techniques, including logistic regression (LR), random forests, gradient boosting trees, convolutional neural networks (CNN), and recurrent neural networks with long short-term memory, training over 100 000 individuals’ longitudinal EHR to predict cardiovascular diseases. The findings highlighted the efficacy of incorporating longitudinal features for enhanced event prediction accuracy. Additionally, integrating genetic features via a late-fusion method was found to significantly enhance cardiovascular disease prediction [14]. They further applied tensor decomposition to model diseases as dynamic processes from longitudinal EHR data, identified, and validated new subphenotypes of cardiovascular diseases [15]. In an unsupervised ML-based method, Sinnott et al. [16] performed clustering to group patients into cases and controls using diagnosis codes. They then calculated the probability of a target phenotype based on the frequency of diagnosis codes. This method exhibited enhanced statistical power for association studies of low-density lipoprotein cholesterol compared to conventional thresholding methods [16]. The availability of large-scale EHR data sources facilitates the training and validation of ML-based phenotyping algorithms. The partnership between RECOVER N3C and the NIH’s All of Us revealed the replicability of N3C’s trained model for long COVID (training set: ∼1 800 000) within the NIH’s All of Us initiative (*n* = ∼300 000). This instance of ML-based phenotype highlights the generalizability of an ML model [17, 18]. While ML approaches do not consistently outperform rule-based algorithms, deep learning (DL) shows comparable performance to traditional ML for multiple conditions [19]. For example, predicting cardiovascular diseases using longitudinal EHR temporal features (e.g. lab values and medications) resulted in comparable performances of area under the receiver operating curve (AUROC) for both ML (0.761–0.790) and CNN (0.784–0.790) approaches [14]. However, generalizing a model to other institutions remains a challenge.

High-throughput phenotyping (HTP), another important phenotyping method, handles hundreds of phenotyping tasks simultaneously. It is typically driven by knowledge, such as dictionaries of diseases and procedure codes. Knowledge-driven HTP (e.g. Phecode [20, 21] and PheMAP [22, 23]) are used widely in large-scale phenotypic analyses. The Phecode method organizes pertinent diagnosis codes into clinically meaningful phenotypes, facilitating researchers in utilizing accumulated ICD data for Phenome-Wide Association Studies (PheWAS) within EHR. While highly efficient, this approach is constrained solely to diagnostic information. To enhance phenotyping accuracy by incorporating additional EHR components [24], PheMAP extends its scope from diagnoses to laboratory results, procedures, prescriptions, and symptoms. A demonstration study exhibits superior performance in Genome-Wide Association Study (GWAS) and PheWAS across various diseases, such as T2DM, dementia, and hypothyroidism, when PheMAP was compared to both Phecode and rule-based algorithms [23]. In particular, there is a hybrid between ML and HTP-based phenotyping algorithms, called PheCAP [25]. The algorithm employs unsupervised feature selection to identify relevant features derived from patients with diagnoses, after which it undergoes training against the gold standard. The performance highly depends on the training dataset.

Other efforts have been established to enable EHR phenotyping using other external non-EHR resources as a starting point, such as online medical content or published biomedical studies [26–29]. These approaches can benefit from innovative methods, including ML, CNN, and Natural Language Processing (NLP), and from standardized medical terminologies and concepts to represent phenotypes. For example, PheMAP [22] is a tool that learns the representation of phenotypes in online consumer health resources, such as the Mayo Clinic Patient Care and Health Information website, MedlinePlus, MedicineNet, Medscape, WikiDoc, and Wikipedia, and they found that online content can provide a reliable resource for phenotyping when compared to EHR-derived approaches [22]. In comparison, published literature can provide a reliable resource for phenotype definitions. Botsis and Ball [30] used abstracts to construct an anaphylaxis semantic network using the co-occurrence of ‘anaphylaxis’ and other medical terms in the same sentence. However, they only addressed one condition, ‘anaphylaxis’, and they did not consider other data in the abstract (e.g. laboratory measurements and the presence of standard codes). Furthermore, they used abstracts rather than full texts, which limited their information retrieval [30].

We hypothesize that biomedical literature is a reliable resource for gathering, harmonizing, and mining information relating to defining a phenotype of interest since many clinical and epidemiological studies publish their phenotype definitions. Our aim in this study is to develop a novel text mining pipeline that retrieves abstracts and full texts that contain phenotype definitions-related information from the literature; to build a classification model to classify phenotype definitions-related sentences from full-text publications and compare the performance between ML and deep learning methods; and to build a large-scale literature knowledgebase of clinical phenotype definitions-related sentences that can be used for future EHR phenotyping applications.

## Materials and methods

### Data sources

Based on findings from a previous work in mining drug–drug interactions [31, 32], a list of 279 clinical phenotypes (Supplementary Appendix A), was proposed after manual review by LL and SQ. For this research, we followed a similar approach to the manual review of literature for abstract selection and full-text retrieval [33]. In selecting abstracts for abstract-level corpus, we used PubMed to search for publications using the 279 clinical phenotypes that were EHR-based studies. Then, we manually reviewed abstracts and classified them as ‘positive = 1’ or ‘negative = 0’. The inclusion criteria for ‘positive = 1’ abstracts are the presence of clinical phenotype definitions information, such as standard codes and the phenotyping inclusion and exclusion criteria. Abstracts that did not meet the ‘positive = 1’ criteria were excluded. For constructing the ‘negative = 0’ abstract set of the corpus, we generated a random sample of PubMed abstracts (1995–2017) using R statistical language.

For full-text sentence-level corpus, we utilized an annotated corpus from our previous work called PhenoDEF [34], which is a corpus aimed to annotate phenotype definitions-related sentences. The sentences were annotated as ‘positive = 1’ if they contained information related to defining a phenotype in EHRs (i.e. phenotyping definitions), which included ‘biomedical and procedure’, ‘standard code’, ‘laboratories and quantitative values’, ‘medication use’, and/or ‘the use of natural language processing’ [34, 35], inspired by PheKB [36]. In addition, sentences with information about data sources (e.g. demographics, vitals, notes, electronic medical records) [37] were used in defining the phenotype we annotated as ‘positive = 1’. Sentences that did not meet the ‘positive = 1’ criteria or included irrelevant information, such as computational and statistical, location, and financial, were annotated as ‘negative = 0’ [34].

### Develop abstract-level classifiers

**Figure 1.**
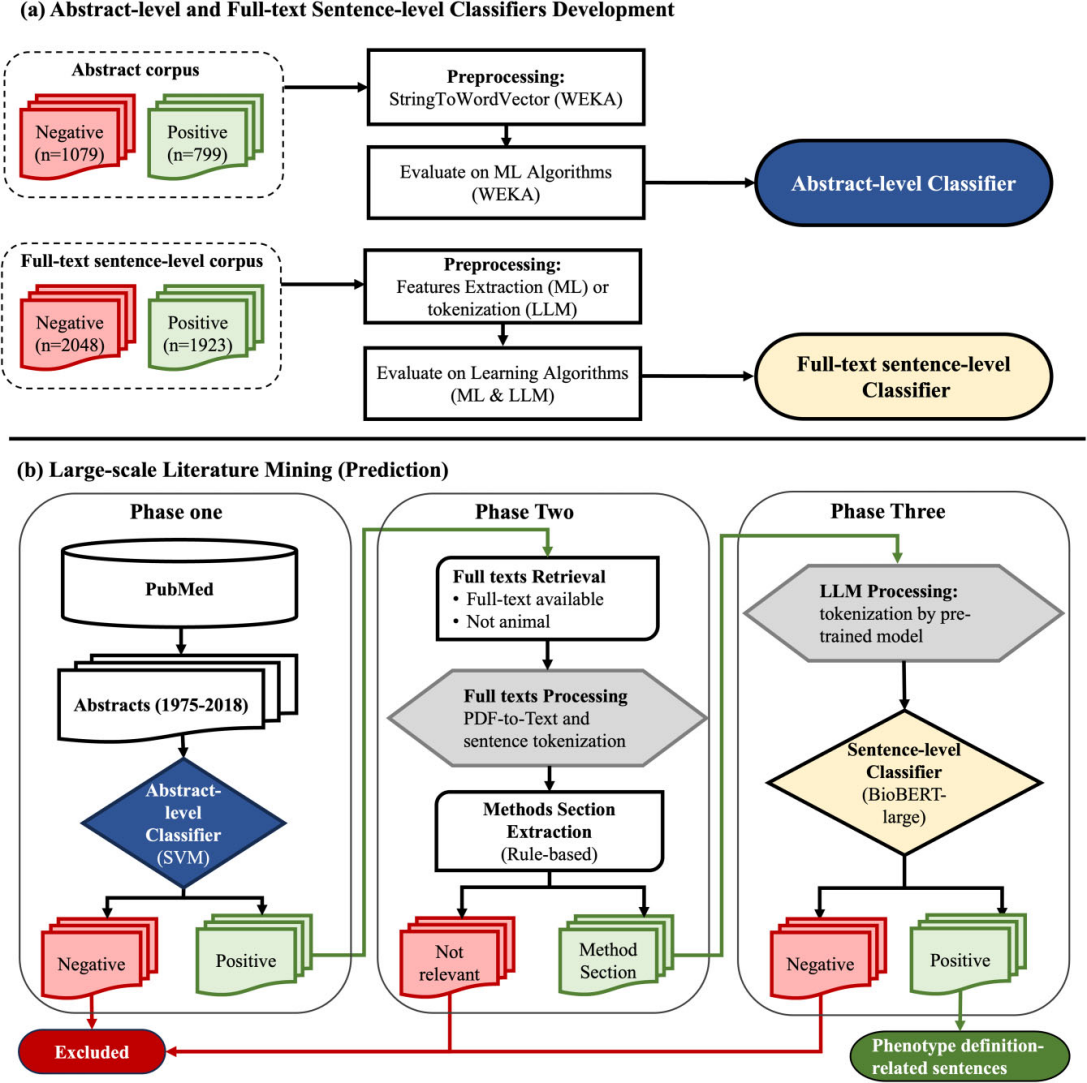
Abstract-level and full-text sentence-level classifiers training and prediction flowchart. (a) Abstract-level and full-text sentence-level classifiers development. (b) Large-scale literature mining (prediction). WEKA, Waikato Environment for Knowledge Analysis; SVM, support vector machine; ML, machine learning; LLM, large language model; BioBERT, Bidirectional Encoder Representations from Transformers for Biomedical Text Mining.

The abstract corpus was read into Waikato Environment for Knowledge Analysis (WEKA) [38] as string attributes where each document contains a title and an abstract (Fig. 1). The ‘StringToWordVector’ module in WEKA to represent each abstract document as a set of attributes, with subspecifications: ‘Lowercase tokens’, ‘WordsToKeep (1000)’, ‘IteratedLovinsStemmer’, ‘StopwordsHandler (MultiStopwords)’, ‘NGramTokenizer (1–3 grams)’, ‘IDFTransform’ (Inverse Document Frequency Transformation), and ‘TFTransform’ (Term frequency score Transformation). We tested the abstract-level classifier on the following ML algorithms: support vector machine (SVM) [sequential minimal optimization (SMO) [39] in WEKA], LR [40], naïve Bayes [41], and decision trees (C4.5 clone [42] called J48 in WEKA). We evaluated them using splits of 70% for training and 30% for testing and reported their accuracy, recall, precision, and F-measure [43].

### Develop full-text sentence-level classifier

For full-text sentence-level classification, we compared the performances of ML, CNN, and large language models (LLMs) when the recall was set to 95%. We utilized the PhenoDEF [34] corpus with binary annotations where sentences containing phenotype definitions-related information are labelled ‘positive = 1’, while others are labelled ‘negative = 0’.

#### ML, feature engineering, and CNN

Before applying ML approach to classify clinical phenotype-related sentences, we began with the feature engineering process. This process involved building a dictionary for named-entity recognition (NER) tasks, designed to represent entities including 279 clinical phenotypes of interest, clinical terms, procedures, and drugs (Table 1).We utilized the Medical Dictionary for Regulatory Activities (MedDRA) [44] for building the initial list of 279 clinical phenotypes in this dictionary (Supplementary Appendix A). To increase the comprehension of our NER dictionary, we further mapped these terms using the exact match method to Systematized Nomenclature of Medicine–Clinical Terms (SNOMED-CT) and Merged Disease Vocabulary (MEDIC) [45]. For the remaining clinical entities, we added terms from MEDIC and SNOMED-CT that were absent from our initial list of 279 clinical phenotypes of interest.

**Table 1.** The named entity recognition (NER) dictionary used for extraction of full-text sentence-level features used in machine learning classifier

Entity and dictionary		Percentage	Number of terms
279 Clinical phenotypes of interest		100%	5627
o	MedDRA PT	4.9%	279
o	SNOMED-CT	62.5%	3517
o	MEDIC (MESH)	32.5%	1831
CLINICAL terms (100%)			471 979
o	SNOMED-CT (body structure, finding, event, observable entity, organism, and situation)	84.3%	398 077
o	MEDIC (MESH)	15.3%	72 167
o	MEDIC (OMIM)	0.4%	1735
Procedures (100%)			190 399
o	SNOMED-CT	98.8%	188 031
o	ICD-9 Procedures	1.2%	2368
Drugs		100%	21 752
o	DrugBank	100%	21 752
Total			689 751

MedDRA PT, Medical Dictionary for Regulatory Activities Preferred Terms; SNOMED-CT, Systematized Nomenclature of Medicine–Clinical Terms; MEDIC, merged disease vocabulary; ICD, International Classification of Diseases.

Following the NER task, new features were generated to represent each sentence as an input matrix of numerical attributes (339 features) as a preparation for the ML algorithms (Supplementary  Table B1). To create and extract features, we adopted a similar approach to the manual annotation process of phenotype definitions described in the PhenoDEF corpus [34], which included phenotype definitions-related features (positive and intermediate evidence) and nonphenotype definitions-related features (negative evidence). We utilized Python to extract these features and to generate the input matrix from all the sentences. These features were represented as the following: Binary features capturing the presence of specific terms or patterns within a sentence, the count of specific terms in a sentence, and the sum of values across multiple features (e.g. the total of several features indicating positive evidence of phenotype definitions-related information). The engineered features represent the sentences based on several text mining approaches, such as single terms or patterns presence, word stemming, co-occurrence of terms or features without considering their order or distance, and regular expression patterns to capture measurable values including blood pressure, laboratory test values, age, height, weight, and body mass index (examples are shown in Supplementary Table B2). We also utilized keywords representing phenotype definitions’ information, including specific words or phrases such as ‘defined’, ‘definition’, ‘classified’, ‘defined as’, ‘identification’, ‘identified’, ‘diagnosis of’, ‘diagnostic criteria’, and ‘case identification’. These keywords were previously identified either manually during the annotation process, or using automated approaches such as n-grams, Term Frequency (TF)-transform, and Inverse Document Frequency (IDF)-Transform [35]. In the example ‘Confirmed adult-onset *asthma (clinical entity)* (AOA) cases were *defined as (definition keyword)* those potential cases with either new-onset *asthma (clinical entity)* or reactivated mild intermittent *asthma (clinical entity)* that had been quiescent for at least one year’ (PMID:12952547), ‘asthma’ was recognized as a clinical entity, and ‘defined as’ was recognized as a definition keyword. Such co-occurrence of clinical entities that were recognized using our NER dictionary (Table 1) and definition keywords served as features for the sentences, indicating that the sentence in this example provides positive evidence of a phenotype definition.

Following the feature engineering process and the generation of the input matrix to represent sentences, we tested the ML classifier using each of the following algorithms: SVM (SMO in WEKA) [39], LR [40], naïve Bayes [41], and decision trees (C4.5 clone [42] called J48 in WEKA). We used WEKA’s manual threshold selector to optimize the LR classifier for high recall (94.2%) for the ‘positive = 1’ class (Supplementary Appendix C). For CNN models, we built a simple CNN model and tuned the parameters (one hidden layer, 200 kernels with size 4). To ensure comparability, we used the same input matrix (339 features) from our feature engineering process to represent sentences in ML and CNN models.

#### Large language models

We also tested Bidirectional Encoder Representations from Transformers (BERT) [46], which is one of the most popular and performed transfer learning techniques. We compared four BERT models, which are BERT-base, BERT-large, Bidirectional Encoder Representations from Transformers for Biomedical Text Mining (BioBERT)-base, and BioBERT-large [47]. All four models were built using the encoder structure from the transformer model but are different in size: The base models consist of 12 layers with 768 multihead attention units per layer, while the large models include 24 layers with 1024 attention units per layer. Compared with the BERT model, which pretrained by general corpus (Wikipedia and BooksCorpus), the BioBERT pretrained by PubMed literature, which made it more specialized in the biomedical domain.

The pretrained BERT model is available online and includes the trained model, a word tokenizer, and a config file. To prepare the data for the BERT model, we converted the sentences using the word tokenizer into numerical representations of each sentence, including three variables: ‘input_ids’, ‘token_type_ids’, and ‘attention_mask’. We set the input length to 512, which is the maximum length that BERT can handle. Sentences longer than 512 were trimmed to keep only the first 512 tokens, and the shorter sentences were padded with zeros to reach a length of 512. We fine-tuned the pretrained model using these vectorized sentences, meaning we continued training it with our labelled samples. During the fine-tuning process, the model maintains its structure settings and parameters and slightly adjusts its weights (learning rate = 5e−5). The fine-tuning process helps the model to learn the specific features for our phenotype definitions mining task, which makes the tokenizer’s representation more suitable for our classification task [46].

### Performance evaluation in clinical phenotype definitions-related sentences classification

We evaluated all models using splits of 70% for training and 30% for testing and reported their accuracy, recall, precision, and F-measure [43]. To achieve a high recall performance in our CNN and LLM models, we added a metric for the accuracy, recall, precision, and F-measure when recall is set at 95% for evaluation. To identify sources of error in the full-text sentence-level classification, we performed an error analysis on 100 randomly selected misclassified sentences and identified possible reasons for their misclassification.

### Generate clinical phenotype definitions-related sentences through implementing abstract-level and full-text sentence-level classifiers to PubMed

#### Phase one – abstract-level classification of abstracts

To start with our screening of abstracts, we downloaded abstracts from PubMed for the years between 1975 and the first quarter of 2018. Prediction of ‘positive = 1’ abstracts was performed using our best ML algorithm, trained and selected from our abstract-level corpus (Fig. 1). All abstracts predicted as ‘positive = 1’ in this phase were further processed into phase two.

#### Phase two – full-text data preprocessing

First, we retrieved the full texts for abstracts predicted as ‘positive = 1’ abstracts. Using our positive set of abstract PMIDs, their PDF and XML formats were downloaded from PubMed if they were open-access articles, or from the subscribed publisher by our institute in case if they were not open access. We excluded abstracts that were not human studies or did not contain full texts. Second, we converted PDF format into text format using the pdftotext tool [48]. Scanned articles (i.e. pictures) and full texts with issues were excluded, as we were not able to convert them into text. In addition, sentences and their boundaries were tokenized using a package called ‘Perl:: Tokenizer’ [49]. Third, we converted text into GENIA [50], an XML format that is used for text mining of the biomedical literature. Within GENIA, the articles were tagged using the PubMed ID, title, and sentences from full texts. We further preprocessed full texts by including sentences within the boundaries of methods section sentences using rule-based methods [51] (Supplementary Appendix D). All relevant sentences in the identified methods section were included in the next phase of classification.

#### Phase three – full-text sentence-level classification

After extracting the methods section relevant sentences, we used our BioBERT-large model for word tokenization and large-scale predictions. The final output from phase three was phenotype definition-related sentences.

### Building a knowledgebase application for the phenotype definitions-related sentences

**Figure 2.**
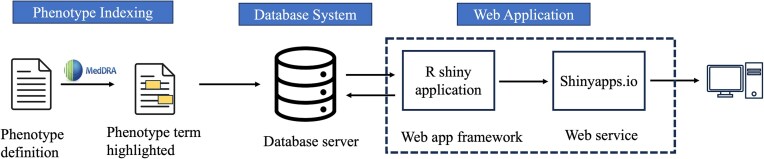
Overview of knowledgebase application for phenotype definitions-related sentences.

To build our phenotype knowledgebase for researchers (Fig. 2), we first utilized MedDRA [44] ‘Preferred Terms’ (PTs) to find any occurrence of these terms within phenotype-related. MedDRA terms cover phenotype information, including diseases, diagnoses, signs, symptoms, qualitative investigation results, indications, and medical/social history [44]. Following the term-matching process, we selectively incorporated sentences containing matched MedDRA PT terms from publicly available articles into the knowledgebase. The application was developed and implemented using the R Shiny framework within the R statistical software (version 4.3.2) [52], connected to the Microsoft Azure-based infrastructure (Microsoft SQL Server). In the current version of the database, all data are organized within a singular table that can be expanded in the future. Dataset attributes (MedDRA_term, term_id, term_start_position, term_end_position, PMID, sentence_index, and sentence_text) were converted into comma-separated values, uploaded to Azure MySQL server, and indexed based on terms for rapid query when using the interface. User inputs are gathered through user interface (UI) elements which are then stored as R variables representing a ‘phenotype of interest’. The option ‘Apply Exact Match’ is used to specify phenotype term boundaries when the user chooses to filter results based on an exact match of terms. Additionally, the feature ‘Filter by Phenotype Keywords’ allows users to add specific keywords (‘ICD’, ‘CPT’, ‘SNOMED’, ‘Laboratory’, and ‘Medication’) as additional filters for the results. The ‘Keywords Boolean Operator’ offers choices (‘OR’, ‘AND’) for users to select the appropriate Boolean operator when making multiple selections of Phenotype Keywords. The outcome of these operations is displayed as tabular results, including PMID, phenotype, and sentence.

The development involved the utilization of several R packages. We used the following R packages in building a knowledgebase application for the phenotype definitions-related sentences: shiny (Functions: shinyApp, page_sidebar, sidebar, checkboxInput, checkboxGroupInput, selectInput, actionButton, downloadButton, updateSelectizeInput, updateCheckboxGroupInput, updateSelectInput, renderText, observeEvent, downloadHandler) [53], shinydashboard (Functions: card, card_header, fluidRow, column) [54], DBI (Function: dbGetQuery) [55], RMariaDB (Function: MariaDB) [56], pool (Functions: dbPool, poolClose) [57], DT (Functions: renderDT, datatable) [58], bslib (Function: bs_theme) [59], tidyverse (Functions: read_csv, select, mutate, filter, reduce, str_detect, str_sub, str_to_lower, as_tibble, map, map_chr) [60], and glue (Function: glue_sql) [61]. We deployed the R Shiny web application on the Shinyapps.io [62] server (https://cliphekb.shinyapps.io/phenotype-main/). The source code is openly available in the GitHub repository: https://github.com/SamarBinkheder/Phenotype_Definitions_App.git.

## Results

The abstract-level and full-text sentence-level corpora were used to train and validate the classifiers (Fig. 1). Table 2 lists the performance results for both classifiers. For the abstract-level classifiers, the SVM and J48 decision tree outperformed the other algorithms with recall, precision, and F-measure of 98%. For the full-text sentence-level classifier, the best results were achieved by BioBERT-large, with 90% accuracy, 95% recall, 87% precision, and 91% F-measure. We identified the possible reasons for misclassification errors in the full-text sentence-level ML classification on 100 randomly selected misclassified sentences (Supplementary Appendix E).

**Table 2.** Abstract-level and full-text sentence-level classifiers performance

Classifiers performance				
Learning algorithm	Accuracy (A @ 95% recall)	Precision (P @ 95% recall)	Recall (R @ 95% recall)	F-measure (F @ 95% recall)
Abstract-level classification				
Support vector machine (SVM)*	0.98	0.98	0.98	0.98
J48 decision tree	0.98	0.98	0.98	0.98
Logistic regression (LR)	0.95	0.95	0.95	0.95
Naïve Bayes	0.96	0.96	0.95	0.95
Full-text sentence-level classification				
Support vector machine (SVM)	0.84	0.84	0.84	0.84
J48 decision tree	0.82	0.82	0.82	0.82
Logistic regression (LR)	0.84	0.84	0.84	0.84
Naïve Bayes	0.82	0.82	0.82	0.82
Convolutional neural network (CNN)	0.88 (0.85)	0.90 (0.79)	0.85 (0.96)	0.87 (0.86)
BERT-base	0.89 (0.89)	0.85 (0.84)	0.95 (0.95)	0.89 (0.89)
BioBERT-base	0.88 (0.88)	0.83 (0.83)	0.95 (0.95)	0.89 (0.89)
BERT-large	0.88 (0.86)	0.83 (0.80)	0.94 (0.95)	0.88 (0.87)
BioBERT-large*	0.90 (0.89)	0.87 (0.85)	0.95 (0.95)	0.91 (0.89)

*The selected algorithm for this classifier.

### Large-scale literature-based mining of phenotyping definitions

We used our validated classifiers for automatic literature-based phenotype definition screening (Table 3). Using the abstract-level classifier, we predicted 459 406 ‘positive = 1’ abstracts, i.e. phenotype definition-related abstracts. For the full-text sentence-level classifier, we used BioBERT-large and predicted 2 580 966 sentences to be ‘positive = 1’, i.e. phenotype definition-related sentences.

**Table 3.** The phenotype knowledge discovery prediction results

Large-scale literature mining prediction results	
Abstract-level classification	
Number of predicted positive abstracts (1975–2018)	459 406
Number of full texts retrieved	141 511
Number of full texts after data processing	120 868
Full-text sentence-level screening	
Total number of sentences (Method section)	5 600 547
Number of predicted positive sentences	2 580 966
Number of predicted negative sentences	3 019 581
MedDRA PT Terms identified in positive sentences	
Number of recognized terms	8031
Number of sentences with recognized terms	1 518 241
Mentions of ICD 9/10, SNOMED, or CPT in positive sentences	
Number of sentences with recognized terms	61 546

MedDRA PT, Medical Dictionary for Regulatory Activities Preferred Terms; ICD, International Classification of Diseases; SNOMED, Systematized Nomenclature of Medicine; CPT, Current Procedural Terminology.

**Figure 3.**
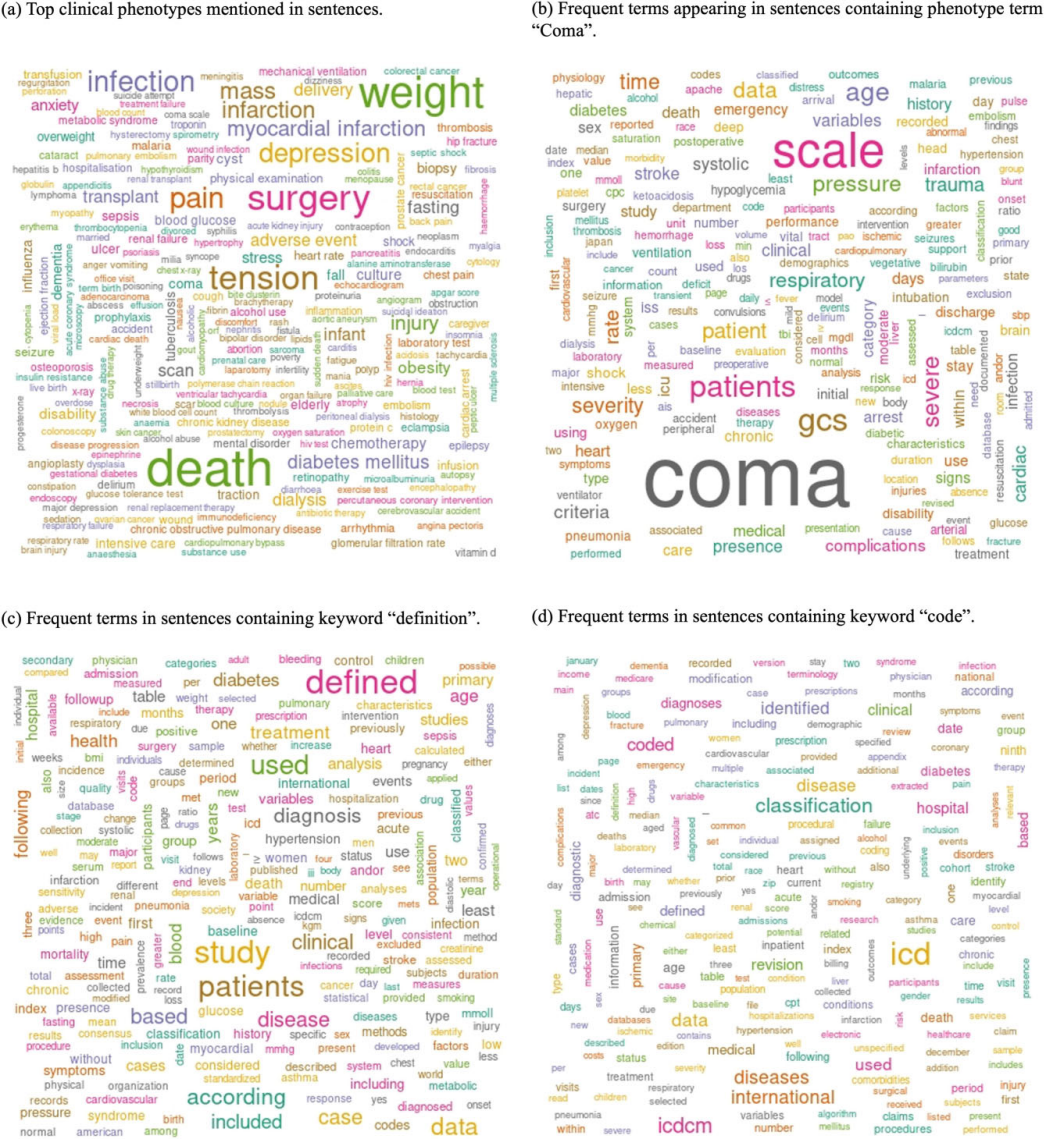
Word cloud analysis of phenotype definition-related sentences, highlighting key terms associated with clinical phenotypes. (a) Top clinical phenotypes mentioned in sentences. (b) Frequent terms appearing in sentences containing the phenotype term ‘Coma’. (c) Frequent terms in sentences containing the keyword ‘definition’. (d) Frequent terms in sentences containing the keyword ‘code’.

Figure 3 shows word cloud representations of phenotypes and sentences classified as phenotype definition-related sentences. We utilized MedDRA terms that were found in the sentences for Wordcloud (Fig. 3a). The top ten terms identified were death (*n* = 89 003), weight (*n* = 74 201), surgery (*n* = 59 972), tension (*n* = 54 068), hypertension (*n* = 46 520), pain (*n* = 44 117), infection (*n* = 43 080), depression (*n* = 38 848), pregnancy (*n* = 33 784), and mass (*n* = 32 029). For the other word clouds, we preprocessed text by converting it to lowercase and removing punctuation, numbers, and stopwords. We then filtered sentences based on the presence of the following keywords: ‘coma’, ‘definition’, and ‘code’ (Fig. 3b–d).

### Clinical Phenotype Knowledgebase

**Figure 4.**
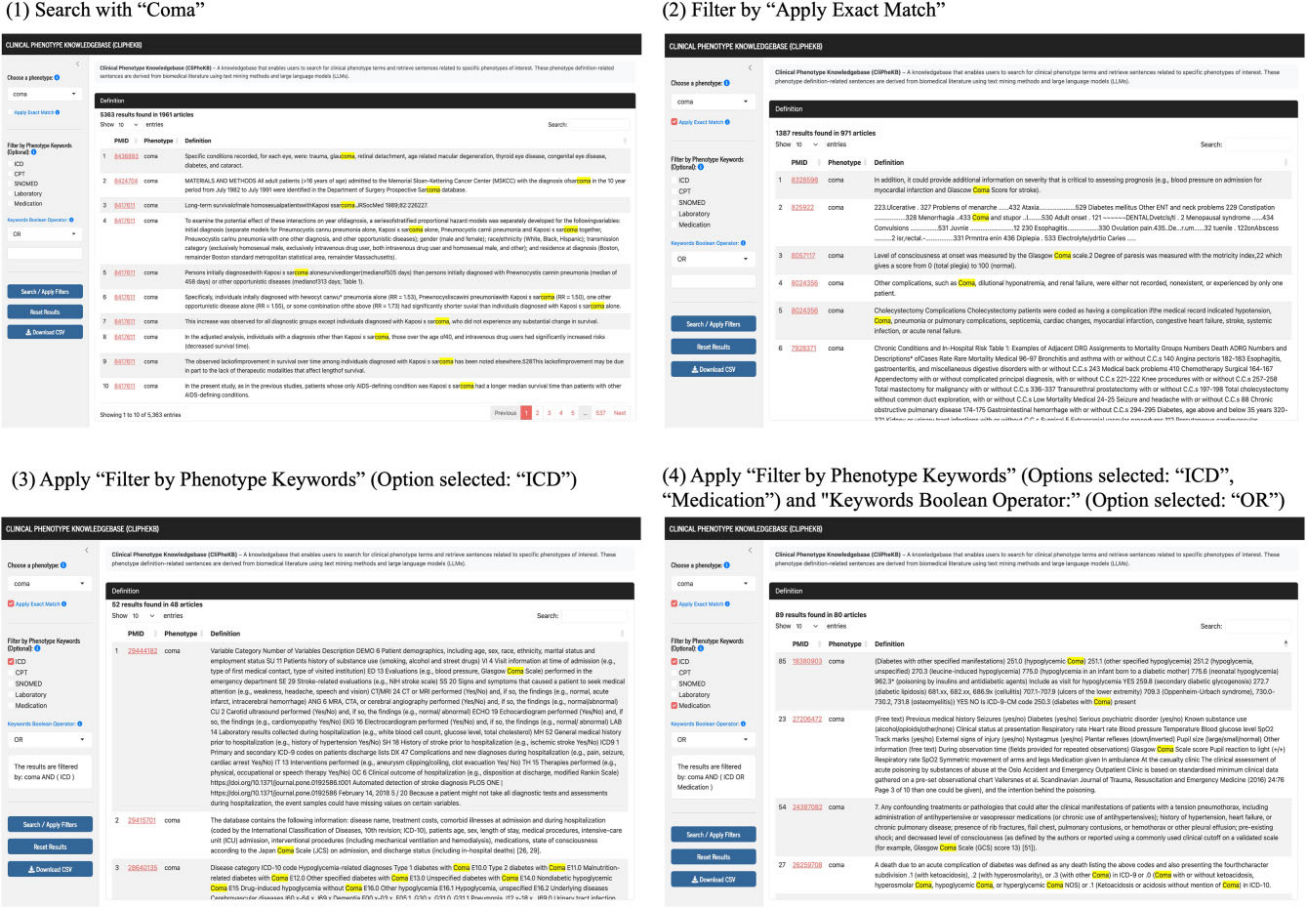
Screenshots of the Clinical Phenotype Knowledgebase (CliPheKB) user interface. Steps 1–4 are shown for ‘Coma’ as an example: (1) Search with ‘Coma’; (2) Filter by ‘Apply Exact Match’; (3) Apply ‘Filter by Phenotype Keywords’ (Option selected: ‘ICD’); and (4) Apply ‘Filter by Phenotype Keywords’ (Options selected: ‘ICD’, ‘Medication’) and ‘Keywords Boolean Operator:’ (Option selected: ‘OR’).

The Clinical Phenotype Knowledgebase (CliPheKB) enables users to search for clinical phenotype terms and retrieve sentences related to a specific phenotype of interest (Fig. 4). Building on the foundation of PheKB [36], we have incorporated filters that allow users to refine their search results using the following phenotype definition keywords: ‘ICD’, ‘CPT’, ‘SNOMED’, ‘Laboratory’, and ‘Medication’. While partial matching of terms within sentences can be beneficial, we also provide users with the option to use an ‘exact match’ filter. This option is particularly useful when the objective is to capture specific terms, such as ‘coma’, without including partially matched terms of ‘coma’, such as ‘glucoma’. Once a term is selected, the interface retrieves results, including the PMID with a link to the corresponding PubMed record for each result, along with the text of the sentences containing the relevant phenotype definitions information. Lastly, the user can download the retrieved results of the search query as a CSV file.

## Discussion

In this study, we compared the performance of ML, CNN, and LLM models in extracting sentences with published phenotype definitions from biomedical literature. Although we began with an initial list of phenotypes of interest, we believe that our approach is designed to be generalizable, as we used scalable text mining techniques to effectively extract literature-based phenotype definition-related sentences. We used biomedical literature for model development, as it provides a standardized, expert-curated, and scalable source of phenotype definitions, enabling robust extraction and minimizing the heterogeneity and bias commonly found in EHR data. Specifically, within full-text articles, we observed that information related to phenotype definitions is frequently found in the methods section of full-text articles. On the other hand, other sections (i.e. introduction, results, and discussion) do not usually contain the exact phenotype definitions used in the study, though sometimes, they provide additional information related to the phenotype definitions, e.g. phenotype names or risk factors associated with the phenotype. We tested several classification algorithms and selected the classifiers with the best performance to ensure coverage of all phenotype-related definition sentences for both abstract-level and full-text sentence-level classification tasks. Specifically, we chose accuracy, precision, recall, and F-measure at 95% recall to ensure nearly complete retrieval of relevant sentences while maintaining high performance metrics. We applied the optimized abstract-level and full-text sentence-level classifiers on large-scale screening of biomedical literature, and we were able to predict over two million sentences with phenotype definition-related sentences. We further developed a knowledgebase called CliPheKB that enables researchers to retrieve phenotype definitions-related sentences from published literature with highlighted phenotypes.

Our efforts to improve the performance of ML, CNN, and LLM classifiers included feature engineering and fine-tuning. Each of these methods has its advantages and disadvantages. The ML approach included an extensive feature engineering process where we engineered these features in a very similar logic to the manual annotation process of these sentences. For example, the features that were closely related to the presence or absence of a phenotype likely brought some performance improvement in the models. NER features that were combined with keywords about ‘definitions’ that indicated a definition of a phenotype might also have improved the model, such as disease names and (‘defined’), which means the co-occurrence of the disease name and the keyword ‘defined’. We validated our ML results by manual review of randomly selected positive sentences, which showed very good coverage of phenotype definitions-related sentences with many false positives, as our aim was to achieve high recall. Compared with ML models, CNN and LLM usually work better in NLP tasks that come at the cost of higher computational resource consumption. We think this is due to the relatively simpler structure of ML methods, which may have a relatively lower ability to catch enough information from the sample and to handle complex NLP problems well. However, we believe that the ML approach performed well during the study, especially when features were effectively engineered. The implementation of CNN and LLM, on the other hand, can offer more comprehensive features with minimal human involvement in the task. Therefore, we believe that CNN and LLM are powerful in identifying sentences containing phenotype definitions-related information when the computational resource is not a limitation.

We evaluated the performance of a simple CNN model and four BERT models in the classification of phenotype definitions-related information. The results showed that the BioBERT-large model outperformed other models. Advantages of using BioBERT include pretrained models requiring minimal sample sizes for fine-tuning and trained on biomedical corpora such as PubMed articles and other medical resources. We believe that this is the key point that made BioBERT a powerful model for classification tasks in this work.

Several areas can be improved in future studies. Future work can include drug information associated with adverse drug reactions from the literature by applying biomedical relation extraction using BioBERT [47]. We used some selected dictionaries and terminologies from the Unified Medical Language System (UMLS); however, we recommend using larger and more comprehensive dictionaries, such as the UMLS Metathesaurus for NLP tasks. In feature engineering tasks, exact matching was used to tag the terms based on prespeculated terminology dictionaries. The exact match methods may be less powerful than other matching methods, and it deserves further investigation. In addition, more sophisticated NLP approaches to recognizing word boundaries are worth exploring [63–65].

Our database offers several potential applications. One application of this database is to recognize the primary phenotype of each sentence. While the current approach recognizes phenotypes within sentences, it does not intentionally distinguish the primary phenotype from other phenotypes mentions within each sentence. The extracted phenotype terms can be further linked to standardized terminologies (e.g. ICD terms and codes), enabling an aggregated representation using standardized terminologies of these definitions for different research purposes. Such future applications can leverage LLMs and generative artificial intelligence to identify primary phenotypes, link them to standardized terminologies and codes, and provide researchers with aggregated and summarized versions of phenotype definitions for each primary phenotype. By providing published phenotype definition-related sentences from biomedical literature, our database can further support automated phenotype algorithm development, reducing reliance on conventional methods and human experts. Integrating it into NLP-based pipelines can further enhance computational phenotype definitions, while combining it with other knowledge sources like standard terminologies and clinical guidelines can also improve these efforts. Additionally, the database can facilitate the extraction of standardized phenotype codes of phenotypes, aiding large-scale cohort studies such as the UK Biobank [66] and All of Us [67], where clinical notes are limited or unavailable [68]. By leveraging this resource, researchers can also enhance cohort identification using structured EHR data such as diagnoses, lab results, and medications [68], reducing manual curation and enabling scalable phenotyping in clinical and genomic research.

This work does not stand without limitations. In developing the corpus, we started the literature query based on 279 selected phenotype terms, and both the abstracts and full-text sentences contain the phenotype terms. While this research began with an initial list of phenotypes, the literature-mined sentence results included not only sentences related to the specified phenotypes but also other sentences associated with additional disease phenotypes. We believe that expanding the corpus to include a broader range of phenotypes could enhance the performance of the classifiers and is worth exploring in future studies. In our NER analyses that created the features for the full-text sentence-level ML model, we used the exact match method. The exact match may lead to many missed terms during the tagging process. Moreover, word boundary detection is one of the most difficult tasks in text mining, and it is more challenging when dealing with multiword concepts or keywords [63–65]. Recognizing abbreviations was challenging. Though we used general approaches to recognize some of the abbreviations, e.g. using term length and preceded or succeeded terms, it is not the scope of this work to fully define and recognize abbreviations. To overcome abbreviations’ limitations in future work, for instance, UMLS’s Specialist Lexicon combined with statistical NLP methods can be used to recognize abbreviations of medical concepts [69]. This is reflected in our error analysis. We therefore applied LLM, which benefits automatic and comprehensive representation of features. Lastly, for CNN, we did not try the CNN with a more complicated structure, i.e. the deeper or wider CNN model, due to the limitation of the labelled sample size. Usually, a CNN model with a more complicated structure may get better performance, but good performance is usually based on a large training set. Considering that we only have a small, labelled dataset, it is hard to say if a more complicated CNN will bring better performance.

## Conclusion

In this work, we developed an innovative approach to mine clinical phenotype definition sentences from published literature. This HTP approach is generalizable and scalable to most published phenotypes, and it is complementary to existing EHR phenotyping methods. The Clinical Phenotype Knowledgebase (CliPheKB) enables users to search for clinical phenotype terms and retrieve sentences related to a specific phenotype of interest.

## Supplementary Material

baaf047_Supplemental_File

## Data Availability

The data underlying this article are available in the article and in its online supplementary material.

## References

[1] Yadav P, Steinbach M, Kumar V, et al. Mining electronic health records (EHRs): a survey. ACM Comput Surv. 2018;50:1–40. 10.1145/3127881

[2] Bakken S . Advancing phenotyping through informatics innovation. J Am Med Inform Assoc. 2023;30:211–12. 10.1093/jamia/ocac247 36651578 PMC9846669

[3] Richesson RL, Hammond WE, Nahm M, et al. Electronic health records based phenotyping in next-generation clinical trials: a perspective from the NIH Health Care Systems Collaboratory. J Am Med Inform Assoc. 2013;20:e226–31. 10.1136/amiajnl-2013-001926 23956018 PMC3861929

[4] Banda JM, Seneviratne M, Hernandez-Boussard T, et al. Advances in electronic phenotyping: from rule-based definitions to machine learning models. Annu Rev Biomed Data Sci. 2018;1:53–68. 10.1146/annurev-biodatasci-080917-013315 31218278 PMC6583807

[5] Carroll RJ, Eyler AE, Denny JC. Naive electronic health record phenotype identification for Rheumatoid arthritis. AMIA Annu Symp Proc. 2011;:189–96. 22195070 PMC3243261

[6] Ferte T, Cossin S, Schaeverbeke T, et al. Automatic phenotyping of electronical health record: pheVis algorithm. J Biomed Inform. 2021;117:103746. 10.1016/j.jbi.2021.103746 33746080

[7] Richesson RL, Marsolo KS, Douthit BJ, et al. Enhancing the use of EHR systems for pragmatic embedded research: lessons from the NIH Health Care Systems Research Collaboratory. J Am Med Inform Assoc. 2021;28:2626–40. 10.1093/jamia/ocab202 34597383 PMC8633608

[8] Newton KM, Peissig PL, Kho AN, et al. Validation of electronic medical record-based phenotyping algorithms: results and lessons learned from the eMERGE network. J Am Med Inform Assoc. 2013;20:e147–54. 10.1136/amiajnl-2012-000896 23531748 PMC3715338

[9] Wei WQ, Denny JC. Extracting research-quality phenotypes from electronic health records to support precision medicine. Genome Med. 2015;7:41. 10.1186/s13073-015-0166-y 25937834 PMC4416392

[10] BHF Data Science Centre . (2023). Ensuring Phenotyping Algorithms Using National Electronic Health Records Are FAIR: Meeting the Needs of the Cardiometabolic Research Community. Zenodo. 10.5281/zenodo.10209724

[11] Kho AN, Hayes MG, Rasmussen-Torvik L, et al. Use of diverse electronic medical record systems to identify genetic risk for type 2 diabetes within a genome-wide association study. J Am Med Inform Assoc. 2012;19:212–18. 10.1136/amiajnl-2011-000439 22101970 PMC3277617

[12] Wei WQ, Li X, Feng Q, et al. LPA variants are associated with residual cardiovascular risk in patients receiving statins. Circulation. 2018;138:1839–49. 10.1161/CIRCULATIONAHA.117.031356 29703846 PMC6202211

[13] Wei WQ, Feng Q, Weeke P, et al. Creation and validation of an EMR-based algorithm for identifying major adverse cardiac events while on statins. AMIA Jt Summits Transl Sci Proc. 2014;2014:112–19. 25717410 PMC4333709

[14] Zhao J, Feng Q, Wu P, et al. Learning from longitudinal data in electronic health record and genetic data to improve cardiovascular event prediction. Sci Rep. 2019;9:717. 10.1038/s41598-018-36745-x 30679510 PMC6345960

[15] Zhao J, Zhang Y, Schlueter DJ, et al. Detecting time-evolving phenotypic topics via tensor factorization on electronic health records: cardiovascular disease case study. J Biomed Inform. 2019;98:103270. 10.1016/j.jbi.2019.103270 31445983 PMC6783385

[16] Sinnott JA, Cai F, Yu S, et al. PheProb: probabilistic phenotyping using diagnosis codes to improve power for genetic association studies. J Am Med Inform Assoc. 2018;25:1359–65. 10.1093/jamia/ocy056 29788308 PMC6915826

[17] Pfaff ER, Girvin AT, Crosskey M, et al. De-black-boxing health AI: demonstrating reproducible machine learning computable phenotypes using the N3C-RECOVER Long COVID model in the All of Us data repository. J Am Med Inform Assoc. 2023;30:1305–12. 10.1093/jamia/ocad077 37218289 PMC10280348

[18] Pfaff ER, Girvin AT, Bennett TD, et al. Identifying who has long COVID in the USA: a machine learning approach using N3C data. Lancet Digit Health. 2022;4:e532–e41. 10.1016/S2589-7500(22)00048-6 35589549 PMC9110014

[19] Yang S, Varghese P, Stephenson E, et al. Machine learning approaches for electronic health records phenotyping: a methodical review. J Am Med Inform Assoc. 2023;30:367–81. 10.1093/jamia/ocac216 36413056 PMC9846699

[20] Wei WQ, Bastarache LA, Carroll RJ, et al. Evaluating phecodes, clinical classification software, and ICD-9-CM codes for phenome-wide association studies in the electronic health record. PLoS One. 2017;12:e0175508. 10.1371/journal.pone.0175508 28686612 PMC5501393

[21] Wu P, Gifford A, Meng X, et al. Mapping ICD-10 and ICD-10-CM codes to phecodes: workflow development and initial evaluation. JMIR Med Inform. 2019;7:e14325. 10.2196/14325 31553307 PMC6911227

[22] Wan NC, Yaqoob AA, Ong HH, et al. Evaluating resources composing the PheMAP knowledge base to enhance high-throughput phenotyping. J Am Med Inform Assoc. 2023;30:456–65. 10.1093/jamia/ocac234 36451277 PMC9933070

[23] Zheng NS, Feng Q, Kerchberger VE, et al. PheMap: a multi-resource knowledge base for high-throughput phenotyping within electronic health records. J Am Med Inform Assoc. 2020;27:1675–87. 10.1093/jamia/ocaa104 32974638 PMC7751140

[24] Wei WQ, Teixeira PL, Mo H, et al. Combining billing codes, clinical notes, and medications from electronic health records provides superior phenotyping performance. J Am Med Inform Assoc. 2016;23:e20–7. 10.1093/jamia/ocv130 26338219 PMC4954637

[25] Zhang Y, Cai T, Yu S, et al. High-throughput phenotyping with electronic medical record data using a common semi-supervised approach (PheCAP). Nat Protoc. 2019;14:3426–44. 10.1038/s41596-019-0227-6 31748751 PMC7323894

[26] Pathak J, Kiefer RC, Chute CG. Using linked data for mining drug–drug interactions in electronic health records. Stud Health Technol. 2013;192:682–86. PMC3909652 23920643

[27] Ning W, Chan S, Beam A, et al. Feature extraction for phenotyping from semantic and knowledge resources. J Biomed Inform. 2019;91:103122. 10.1016/j.jbi.2019.103122 30738949 PMC6424621

[28] Yu S, Liao KP, Shaw SY, et al. Toward high-throughput phenotyping: unbiased automated feature extraction and selection from knowledge sources. J Am Med Inform Assoc. 2015;22:993–1000. 10.1093/jamia/ocv034 25929596 PMC4986664

[29] Yu S, Chakrabortty A, Liao KP, et al. Surrogate-assisted feature extraction for high-throughput phenotyping. J Am Med Inform Assoc. 2016;24:e143–e9. 10.1093/jamia/ocw135 PMC6080726 27632993

[30] Botsis T, Ball R. Automating case definitions using literature-based reasoning. Appl Clin Inform. 2013;4:515–27. 24454579 10.4338/ACI-2013-04-RA-0028 PMC3885912

[31] Duke JD, Han X, Wang Z, et al. Literature based drug interaction prediction with clinical assessment using electronic medical records: novel myopathy associated drug interactions. PLoS Comput Biol. 2012;8:e1002614. 10.1371/journal.pcbi.1002614 22912565 PMC3415435

[32] Wu HY, Zhang S, Desta Z, et al. Translational drug interaction evidence gap discovery using text mining. Clin Pharmacol Ther. 2017;101:S91–S2.

[33] Cohen AM, Adams CE, Davis JM, et al. Evidence-based medicine, the essential role of systematic reviews, and the need for automated text mining tools. In: Proceedings of the 1st ACM International Health Informatics Symposium, Arlington, VA, 376–80.

[34] Binkheder S, Wu HY, Quinney SK et al. PhenoDEF: a corpus for annotating sentences with information of phenotype definitions in biomedical literature. J Biomed Semantics. 2022;13:17. 10.1186/s13326-022-00272-6 35690873 PMC9188713

[35] Binkheder S, Wu H-Y, Quinney S, Li L (eds). Analyzing patterns of literature-based phenotyping definitions for text mining applications. In: IEEE International Conference on Healthcare Informatics (ICHI), 4–7 June, 2018.

[36] Kirby JC, Speltz P, Rasmussen LV, et al. PheKB: a catalog and workflow for creating electronic phenotype algorithms for transportability. J Am Med Inform Assoc. 2016;23:1046–52. 10.1093/jamia/ocv202 27026615 PMC5070514

[37] Shivade C, Raghavan P, Fosler-Lussier E, et al. A review of approaches to identifying patient phenotype cohorts using electronic health records. J Am Med Inform Assoc. 2014;21:221–30. 10.1136/amiajnl-2013-001935 24201027 PMC3932460

[38] Frank E, Hall M, Holmes G, et al. Weka: a machine learning workbench for data mining. In: Maimon, Rokach (eds.), Data Mining and Knowledge Discovery Handbook, 2nd edn. Boston, MA: Springer, 2010, 1269–77.

[39] Platt JC . Fast training of support vector machines using sequential minimal optimization. Advances in kernel methods. 1999;:185–208.

[40] Quinlan JR . C4. 5: Programs for Machine Learning. New York, NY: Elsevier, 2014.

[41] John GH, Langley P. Estimating continuous distributions in Bayesian classifiers. In: Proceedings of the 11th Conference on Uncertainty in Artificial Intelligence, Canada, San Mateo, CA: Morgan Kaufmann Publishers Inc, 1995, 338–45.

[42] Lecessie S, Vanhouwelingen JC. Ridge estimators in logistic-regression. J R Stat Soc Ser C. 1992;41:191–201.

[43] Zaki MJ, Meira W Jr, Meira W. Data Mining and Analysis: Fundamental Concepts and Algorithms. Cambridge: Cambridge University Press, 2014.

[44] Mozzicato P . MedDRA. Pharm Med. 2009;23:65–75. 10.1007/BF03256752

[45] Davis AP, Wiegers TC, Rosenstein MC et al. MEDIC: a practical disease vocabulary used at the Comparative Toxicogenomics Database. Database. 2012;2012:bar065. 10.1093/database/bar065 22434833 PMC3308155

[46] Devlin J, Chang MW, Lee K, Toutanova K. BERT: pre-training of deep bidirectional transformers for language understanding. In: Proceedings of the Conference of NAACL HLT 2019 Conference of the North American Chapter of the Association for Computational Linguistics: Human Language Technologies. 1, 2018, 4171–86.

[47] Lee J, Yoon W, Kim S, et al. BioBERT: a pre-trained biomedical language representation model for biomedical text mining. Bioinformatics. 2020;36:1234–40. 10.1093/bioinformatics/btz682 31501885 PMC7703786

[48] Noonburg D . Xpdf and XpdfReader 1995. http://www.xpdfreader.com/about.html (1 October 2023, date last accessed).

[49] Perl::Tokenizer—A tiny Perl code tokenizer.—metacpan.org.

[50] Kim JD, Ohta T, Tateisi Y, et al. GENIA corpus—semantically annotated corpus for bio-textmining. Bioinformatics. 2003;19:i180–2. 10.1093/bioinformatics/btg1023 12855455

[51] Agarwal S, Yu H. Automatically classifying sentences in full-text biomedical articles into Introduction, Methods, Results and Discussion. Bioinformatics. 2009;25:3174–80. 19783830 10.1093/bioinformatics/btp548 PMC2913661

[52] The R Project for Statistical Computing . https://www.r-project.org/ (12 January 2024, date last accessed).

[53] Shiny. https://shiny.posit.co/ (12 January 2024, date last accessed).

[54] Shiny Dashboard. https://rstudio.github.io/shinydashboard/ (12 January 2024, date last accessed).

[55] DBI: R Database Interface. https://cran.r-project.org/web/packages/DBI/index.html (12 January 2024, date last accessed).

[56] RMariaDB: Database Interface and MariaDB Driver. https://cran.r-project.org/web/packages/RMariaDB/index.html (12 January 2024, date last accessed).

[57] Pool: Object Pooling. https://cran.r-project.org/web/packages/pool/index.html (12 January 2024, date last accessed).

[58] DT: A Wrapper of the JavaScript Library ‘DataTables’. https://cran.r-project.org/web/packages/DT/index.html (12 January 2024, date last accessed).

[59] Bslib: Custom ‘Bootstrap’ ‘Sass’ Themes for ‘shiny’ and ‘rmarkdown’. https://cran.r-project.org/web/packages/bslib/index.html (12 January 2024, date last accessed).

[60] Tidyverse: Easily Install and Load the ‘Tidyverse’. https://cran.r-project.org/web/packages/tidyverse/index.html (12 January 2024, date last accessed).

[61] Glue: Interpreted String Literals. https://cran.r-project.org/web/packages/glue/index.html (12 January 2024, date last accessed).

[62] Shinyapps.io. https://www.shinyapps.io/ (12 January 2024, date last accessed).

[63] Chang JT, Schutze H, Altman RB. GAPSCORE: finding gene and protein names one word at a time. Bioinformatics. 2004;20:216–25. 10.1093/bioinformatics/btg393 14734313

[64] Krauthammer M, Nenadic G. Term identification in the biomedical literature. J Biomed Inform. 2004;37:512–26. 10.1016/j.jbi.2004.08.004 15542023

[65] Wang X, Yang C, Guan RC. A comparative study for biomedical named entity recognition. Int J Mach Learn Cyb. 2018;9:373–82. 10.1007/s13042-015-0426-6

[66] Bycroft C, Freeman C, Petkova D, et al. The UK Biobank resource with deep phenotyping and genomic data. Nature. 2018;562:203–209. 10.1038/s41586-018-0579-z 30305743 PMC6786975

[67] The All of Us Research Program Investigators . The ‘All of Us’ Research Program. N Engl J Med. 2019;381:668–76. 10.1056/NEJMsr1809937 31412182 PMC8291101

[68] Castro VM, Minnier J, Murphy SN, et al. Validation of electronic health record phenotyping of bipolar disorder cases and controls. Am J Psychiatry. 2015;172:363–72. 10.1176/appi.ajp.2014.14030423 25827034 PMC4441333

[69] Moon S, Pakhomov S, Liu N, et al. A sense inventory for clinical abbreviations and acronyms created using clinical notes and medical dictionary resources. J Am Med Inform Assoc. 2014;21:299–307. 10.1136/amiajnl-2012-001506 23813539 PMC3932450

