# Supplementary material for: Biomedical literature-based clinical phenotype definition discovery using large language models
Source: Database (Oxford). 2025 Sep 24;2025:baaf047. doi: 10.1093/database/baaf047 (PMC12462612; doi:10.1093/database/baaf047)
Supplement: baaf047_Supplemental_File [file baaf047_supplemental_file.docx]

**Supplementary Appendices**

Table of Contents

[Appendix A – Clinical Phenotypes 2](#_Toc200446628)

[Appendix B – Feature engineering for machine learning (ML)’s full-text sentence-level classifier 6](#_Toc200446629)

[Appendix C – Full-text sentence-level classifier performance using logistic regression across different thresholds for the “positive” class. 8](#_Toc200446630)

[Appendix D – IMRAD keywords and rules 9](#_Toc200446631)

[Appendix E – Error analysis of the machine learning classifier 10](#_Toc200446632)

[References 12](#_Toc200446633)

Appendix A – Clinical Phenotypes

**Table A1.** The list of 279 clinical phenotypes of interest

| **Phenotypes (1-150)** | **MedDRA PT** | **Phenotypes (151-279)** | **MedDRA PT** |
| --- | --- | --- | --- |
| Abscess | 10000269 | Insomnia | 10022437 |
| Acne | 10000496 | Irritability | 10022998 |
| Acute coronary syndrome | 10051592 | Ischaemia | 10061255 |
| Affect lability | 10054196 | Ischaemic stroke | 10061256 |
| Aggression | 10001488 | Jaundice | 10023126 |
| Agitation | 10001497 | Lethargy | 10024264 |
| Akathisia | 10001540 | Leukocytosis | 10024378 |
| Alopecia | 10001760 | Leukocyturia | 10050791 |
| Anaemia | 10002034 | Leukopenia | 10024384 |
| Aneurysm | 10002329 | Lipoatrophy | 10024604 |
| Angina pectoris | 10002383 | Lipodystrophy acquired | 10049287 |
| Anxiety | 10002855 | Liver disorder | 10024670 |
| Anxiety disorder | 10057666 | Liver injury | 10067125 |
| Arrhythmia | 10003119 | Lung disorder | 10025082 |
| Arteriosclerosis | 10003210 | Lymphocele | 10048642 |
| Arthritis | 10003246 | Lymphoproliferative disorder | 10061232 |
| Asthenia | 10003549 | Malaise | 10025482 |
| Asthma | 10003553 | Mania | 10026749 |
| Ataxia | 10003591 | Menorrhagia | 10027313 |
| Atrial fibrillation | 10003658 | Methaemoglobinaemia | 10027496 |
| Atrioventricular block | 10003671 | Miosis | 10027646 |
| Atrioventricular block second degree | 10003677 | Mitochondrial toxicity | 10053961 |
| Azotaemia | 10003885 | Multi-organ failure | 10028154 |
| Back pain | 10003988 | Muscular weakness | 10028372 |
| Bipolar disorder | 10057667 | Musculoskeletal pain | 10028391 |
| Blood cholesterol increased | 10005425 | Musculoskeletal stiffness | 10052904 |
| Blood creatinine increased | 10005483 | Mutism | 10028403 |
| Blood pressure decreased | 10005734 | Myalgia | 10028411 |
| Bone marrow failure | 10065553 | Myocardial infarction | 10028596 |
| Bradycardia | 10006093 | Myocardial ischaemia | 10028600 |
| Bundle branch block left | 10006580 | Myoclonus | 10028622 |
| Cachexia | 10006895 | Myopathy | 10028641 |
| Cardiac arrest | 10007515 | Myositis | 10028653 |
| Cardiac failure | 10007554 | Nail disorder | 10028694 |
| Cardiac failure congestive | 10007559 | Nephrolithiasis | 10029148 |
| Cardiac fibrillation | 10061592 | Nephropathy | 10029151 |
| Cardiomegaly | 10007632 | Nephropathy toxic | 10029155 |
| Cardiotoxicity | 10048610 | Nephrotic syndrome | 10029164 |
| Cerebrovascular accident | 10008190 | Nervousness | 10029216 |
| Chest discomfort | 10008469 | Neuralgia | 10029223 |
| Chills | 10008531 | Neuropathy peripheral | 10029331 |
| Cholelithiasis | 10008629 | Neurotoxicity | 10029350 |
| Cholestasis | 10008635 | Neutropenia | 10029354 |
| Chronic allograft nephropathy | 10063209 | Nightmare | 10029412 |
| Cognitive disorder | 10057668 | Obsessive-compulsive disorder | 10029898 |
| Coma | 10010071 | Oedema | 10030095 |
| Completed suicide | 10010144 | Oliguria | 10030302 |
| Confusional state | 10010305 | Osteopenia | 10049088 |
| Constipation | 10010774 | Overdose | 10033295 |
| Convulsion | 10010904 | Pain | 10033371 |
| Coronary artery disease | 10011078 | Palpitations | 10033557 |
| Cough | 10011224 | Pancreatitis | 10033645 |
| Crying | 10011469 | Pancytopenia | 10033661 |
| Cyanosis | 10011703 | Panic attack | 10033664 |
| Delirium | 10012218 | Panic disorder | 10033666 |
| Delusion | 10012239 | Paraesthesia oral | 10057372 |
| Dementia | 10012267 | Parkinsonism | 10034010 |
| Depression | 10012378 | Peptic ulcer | 10034341 |
| Dermatitis | 10012431 | Peripheral sensory neuropathy | 10034620 |
| Diabetes mellitus | 10012601 | Peripheral vascular disorder | 10034636 |
| Diarrhoea | 10012735 | Pharyngitis | 10034835 |
| Dissociation | 10013457 | Poisoning | 10061355 |
| Dizziness | 10013573 | Polyuria | 10036142 |
| Drug intolerance | 10061822 | Poor quality sleep | 10062519 |
| Drug tolerance | 10052804 | Pregnancy | 10036556 |
| Drug tolerance decreased | 10052805 | Presyncope | 10036653 |
| Dry mouth | 10013781 | Productive cough | 10036790 |
| Duodenal ulcer | 10013836 | Proteinuria | 10037032 |
| Dysarthria | 10013887 | Prothrombin time prolonged | 10037063 |
| Dyslipidaemia | 10058108 | Pruritus | 10037087 |
| Dysphagia | 10013950 | Psoriasis | 10037153 |
| Dyspnoea | 10013968 | Psychosomatic disease | 10049587 |
| Dystonia | 10013983 | Psychotic disorder | 10061920 |
| Electrocardiogram qt interval | 10014385 | Pulmonary toxicity | 10061924 |
| Electrocardiogram qt prolonged | 10014387 | Pyelonephritis | 10037596 |
| Electrocardiogram st segment | 10014389 | Rash | 10037844 |
| Embolism | 10061169 | Renal failure | 10038435 |
| Epistaxis | 10015090 | Renal failure chronic | 10038444 |
| Erectile dysfunction | 10061461 | Renal impairment | 10062237 |
| Erythema | 10015150 | Renal tubular necrosis | 10038540 |
| Erythema multiforme | 10015218 | Restlessness | 10038743 |
| Essential hypertension | 10015488 | Rhabdomyolysis | 10039020 |
| Euphoric mood | 10015535 | Rhinitis | 10039083 |
| Extrapyramidal disorder | 10015832 | Salivary hypersecretion | 10039424 |
| Fatigue | 10016256 | Schizoaffective disorder | 10039621 |
| Fluid retention | 10016807 | Schizophrenia | 10039626 |
| Flushing | 10016825 | Sedation | 10039897 |
| Formication | 10017062 | Serotonin syndrome | 10040108 |
| Gait disturbance | 10017577 | Sexual dysfunction | 10040477 |
| Gastric ulcer | 10017822 | Shock | 10040560 |
| Gastrointestinal haemorrhage | 10017955 | Sinus bradycardia | 10040741 |
| Gastrooesophageal reflux disease | 10017885 | Sinusitis | 10040753 |
| Gingival hyperplasia | 10018283 | Skin toxicity | 10059516 |
| Glomerulonephritis | 10018364 | Sleep disorder | 10040984 |
| Glucose tolerance impaired | 10018429 | Social avoidant behaviour | 10041243 |
| Glycosuria | 10018473 | Somnolence | 10041349 |
| Gout | 10018627 | Stomatitis | 10042128 |
| Graft dysfunction | 10059677 | Stress | 10042209 |
| Graft loss | 10048748 | Sudden cardiac death | 10049418 |
| Graft versus host disease | 10018651 | Sudden death | 10042434 |
| Grand mal convulsion | 10018659 | Suicidal ideation | 10042458 |
| Gynaecomastia | 10018800 | Suicide attempt | 10042464 |
| Haematoma | 10018852 | Syncope | 10042772 |
| Haematuria | 10018867 | Tachycardia | 10043071 |
| Haemolysis | 10018910 | Tardive dyskinesia | 10043118 |
| Haemorrhage | 10055798 | Tension | 10043268 |
| Haemorrhagic diathesis | 10062713 | Thinking abnormal | 10043431 |
| Hallucination | 10019063 | Thrombocytopenia | 10043554 |
| Hemiparesis | 10019465 | Thrombosis | 10043607 |
| Hemiplegia | 10019468 | Thrombotic thrombocytopenic purpura | 10043648 |
| Hepatic cirrhosis | 10019641 | Torsade de pointes | 10044066 |
| Hepatic encephalopathy | 10019660 | Transaminases increased | 10054889 |
| Hepatic enzyme increased | 10060795 | Tremor | 10044565 |
| Hepatic failure | 10019663 | Type 2 diabetes mellitus | 10067585 |
| Hepatic function abnormal | 10019670 | Ulcer | 10045285 |
| Hepatic steatosis | 10019708 | Upper gastrointestinal haemorrhage | 10046274 |
| Hepatitis cholestatic | 10019754 | Urinary incontinence | 10046543 |
| Hepatotoxicity | 10019851 | Urticaria | 10046735 |
| High density lipoprotein decreased | 10020060 | Vasoconstriction | 10047139 |
| Hostility | 10020400 | Ventricular arrhythmia | 10047281 |
| Hot flush | 10060800 | Ventricular extrasystoles | 10047289 |
| Hyperbilirubinaemia | 10020578 | Ventricular failure | 10060953 |
| Hypercalcaemia | 10020583 | Ventricular fibrillation | 10047290 |
| Hyperchlorhydria | 10020601 | Ventricular tachycardia | 10047302 |
| Hypercholesterolaemia | 10020603 | Vision blurred | 10047513 |
| Hyperglycaemia | 10020635 | Visual impairment | 10047571 |
| Hyperhidrosis | 10020642 | Weight decreased | 10047895 |
| Hyperkalaemia | 10020646 | Weight increased | 10047899 |
| Hyperlipidaemia | 10062060 | Withdrawal syndrome | 10048010 |
| Hypersensitivity | 10020751 |  |  |
| Hypertension | 10020772 |  |  |
| Hyperthyroidism | 10020850 |  |  |
| Hypertriglyceridaemia | 10020869 |  |  |
| Hypertrophic cardiomyopathy | 10020871 |  |  |
| Hyperuricaemia | 10020903 |  |  |
| Hypoalbuminaemia | 10020942 |  |  |
| Hypochondriasis | 10020965 |  |  |
| Hypoglycaemia | 10020993 |  |  |
| Hypokalaemia | 10021015 |  |  |
| Hypomagnesaemia | 10021027 |  |  |
| Hypomania | 10021030 |  |  |
| Hyponatraemia | 10021036 |  |  |
| Hypophosphataemia | 10021058 |  |  |
| Hypoprothrombinaemia | 10021085 |  |  |
| Hypotension | 10021097 |  |  |
| Hypothyroidism | 10021114 |  |  |
| Idiopathic thrombocytopenic purpura | 10021245 |  |  |
| Immunodeficiency | 10061598 |  |  |
| Incontinence | 10021639 |  |  |
| Infarction | 10061216 |  |  |

Appendix B – Feature engineering for machine learning (ML)’s full-text sentence-level classifier

**Table B1.** Full-text sentence-level machine learning features.

| **Feature Type** | **Description** | **Variable Type** | **Count** | **Example Features** |
| --- | --- | --- | --- | --- |
| Phenotype definitions-related features with positive evidence | Features suggest the presence of information related to phenotype definitions in a sentence. | Binary | 304 | - CLINICAL_NER - Procedure_NER - lab_values_regex |
|  |  | Non-binary (Counts of terms) | 5 | - Defintion_term_count - Code_names - NLP_words |
|  |  | Non-binary (sum of columns with positive evidence) | 1 | - Sum_inclusion_columns |
| Phenotype definitions-related features with intermediate evidence | Features do not show direct evidence of a phenotype definition but could assist by providing supporting evidence for phenotyping (intermediate evidence). | Binary | 9 | - Record_AND_Review - Manual_AND_Review |
|  |  | Non-binary (Counts of terms) | 6 | - Medical_records - Study_design |
|  |  | Non-binary (sum of columns with intermediate evidence) | 1 | - Sum_inter_columns |
| Non-phenotype definitions-related features with negative evidence | Features represent sentences that are out of the scope of a phenotype definition or phenotyping (negative evidence). | Binary | 10 | - Location - Financial - Software_or_tool |
|  |  | Non-binary (Counts of terms) | 1 | - Statistical |
|  |  | Non-binary (sum of columns with negative evidence) | 1 | - Sum_exclu_columns |

**Table B2.** Examples of sentences used for feature engineering.

| Description | Semantic relationship | Example |
| --- | --- | --- |
| **Patterns** | Regular expression to capture lab values | “diabetes was diagnosed if a patient had fasting plasma glucose of 126 mg/dl or greater, or a random glucose greater than 200 mg/dl” (PMID20819866) |
| **Single term (complete or stemmed)** | Defin (definition keywords) | “Confirmed adult-onset asthma (AOA) cases were defined as those potential cases with either new-onset asthma or reactivated mild intermittent asthma that had been quiescent for at least one year.” (PMID12952547) |
|  | code | “Cerebrovascular disease was defined as the presence of ICD-9-CM diagnosis codes 430. X X -438. X X” (PMID20819866) |
| **Phrases** | Evidence of (definition keywords) | Controls were patients without evidence of PAD. (PMID20819866) |
|  | Medical records (Data used) | “We retrospectively reviewed the medical records to collect the following data: patient age, sex, smoking history, previous and current antihypertensive medications, history of intolerance to antihypertensive agents, comorbid conditions, and BP.” (PMID11388131) |
| **Medical NER presence** | CLINICAL | “patients for hyperkalemia: (1) potassium value &gt;5.5 mmol/L; or (2) diagnosis code for hyperkalemia.” (PMID17712071) |
|  |  | “Patient has heart disease diagnosis at any time.” (PMID23449283) |
|  | Procedure | “The primary endpoint was an asthma-related event (ARE), which was defined as (1) an emergency department (ED) visit or (2) hospital admission with a primary asthma diagnosis code ICD-9-CM code 493.xx.” (PMID17269833) |
| **Two words co-occurrence** | Definition keywords + Medical NER Presence | “target BP was defined as systolic BP” (PMID11388131) |
|  | Inclusion criteria terms (definition keywords) + Exclusion criteria terms (definition keywords) | “the inclusion and exclusion criteria of the clinical definition were mapped to a list of ICD9CM codes.” (PMID27940627) |
|  | Inclusion terms (definition keywords) + Patient terms | “Patients with the presence of at least one diagnosis of major depression determined . . . from the EMR for inclusion in a data set (referred to as a data mart).” (PMID22466034) |
| **Two words co-occurrence followed by abbreviation (with order)** | Patient terms + with + abbreviation | “Patients with CAD (as defined by a history of myocardial infarction . . .” (PMID16765240) |
| **Co-occurrence of terms preceded with abbreviation (with order)** | Abbreviation + [diagno \| event \| disease \| identif (definition keywords)] | “We used the confirmed SCD diagnosis from Michigan NBS administrative records as the gold standard.” (PMID24882379) |
|  | Abbreviation + [positive \| negative] | “HF positive vs HF negative.” (PMID17567225) |

Appendix C – Full-text sentence-level classifier performance using logistic regression across different thresholds for the “positive” class.


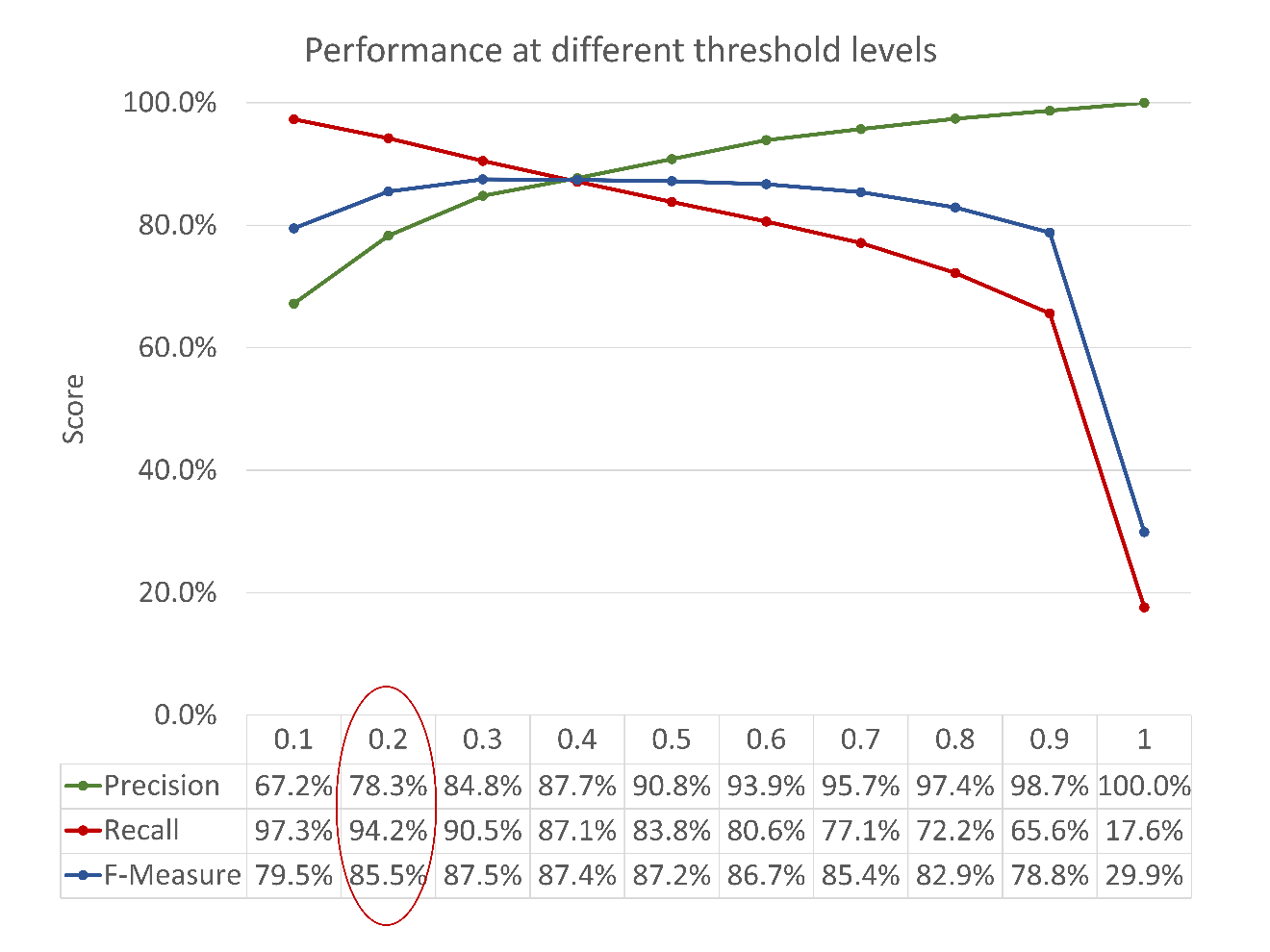


**Figure C1.** Full-text sentence-level classifier performance using logistic regression across different thresholds for the “positive” class.

Figure C1 shows the plot to visualize the threshold values of the predicted probability for logistic regression (LR). The default threshold that is used in Weka is 0.5 where the predicted probability should be higher than 0.5 to be predicted as “positive”. This threshold can be adjusted manually in Weka using “manualThresholdValue” for values between 0 and 1. Since we were interested in increasing the recall, we selected 0.2 as our threshold for ‘positive’ category with a recall of 94.2%.

Appendix D – IMRAD keywords and rules

**Table D1.** IMRAD keywords and rules

| **Keywords used to identify methods sections** | |
| --- | --- |
| Section | Keywords |
| Relevant (Method section) | "Methodology", "METHODS", "Methods", "Method", "METHOD","DESIGN, SETTING, AND PATIENTS", "Design, Setting, and Participants", "Design", "DESIGN", "SETTING", "Setting", "SUBJECTS", "Materials and methods", "Materials and Methods", "Material and methods", "Patients and methods", "Participants and methods", "Experiment", "EXPERIMENT", "Subjects and methods", "Data source", "Research design and methods", "Methods and Procedures", "Methods and Materials“ |
| Not relevant | "Discussion", "DISCUSSION", "Findings", "Finding", "Result", "RESULT", "Results", "FINDING", "BACKGROUND", "Backgrouand", "Introduction", "INTRODUCTION", "IMPORTANCE", "Keywords", "Key Words:", "In conclusion", "Conclusion", "CONCLUSION", "REFERENCES", "COMMENT" |
| **Examples of rules used** | |
| - A sentence starts with a keyword - "part 1: CHECK IF IT ENDS WITH S" - "part 2: CHECK IF IT FOLLOWED BY : OR ." - "part 3: CHECK IF IT FOLLOWED BY SPACE" - "part 4: CHECK IF IT ENDS WITH —" - Check if the following word is upper case or number - The position of the sentence | |

Abbreviations: Introduction, Method, Results and Discussion (IMRAD)

Appendix E – Error analysis of the machine learning classifier

We identified the possible reasons for misclassification errors in the full-text sentence-level machine learning (ML) classification on 100 randomly selected misclassified sentences (Table E1). Ambiguity is one of the text mining challenges where in some situations it was hard to infer the meaning of a character, symbol, or term. For example, periods can indicate the end of a sentence or a word abbreviation leading to inappropriate sentence segmentation [1]. Clinical and keyword dictionaries showed error percentages of 37% and 35%, respectively. Word boundary detection and semantic ambiguity were the least frequent errors in this subset of 100 sentences. Negative atypical means that either a negative sentence shows cues similar to a positive sentence or it has insufficient negative cues. In contrast, positive atypical sentences show no or insufficient evidence of positive cues for phenotyping. These sentences provide supportive information for phenotyping; however, they can be too short to provide evidence. The clinical dictionary refers to clinical and medical entities from existing standardized dictionaries, whereas the keywords refers to terms that we derived or proposed based on our corpus analysis of positive or/and negative phenotyping definitions cues contained within sentences.

**Table E1.** Examples of errors identified.

| **Error** | **Example** | **Positive (n=50)** | **Negative (n=50)** | **Total (n=100)** |
| --- | --- | --- | --- | --- |
| Abbreviations | “A little more than one third of all patients identified by the NLP method were manually confirmed to have HF.” (PMID17567225)  HF was not recognized as a clinical phenotype in the sentence. | 27(54%) | 3 (6%) | 30 (30%) |
| Word boundary detection | “First-line treatment.” (PMID11388131)  This sentence is an actual negative and predicted as positive. “men” is one of the keywords of patient. Here, "men" recognized from "treatment" due to incorrect word boundary identification. | 4(8%) | 12(24%) | 16 (16%) |
| Semantic ambiguity | “Since nearly everyone residing in the target ZIP code for the current study receives their health care through Marshfield Clinic, this record is considered comprehensive.” (PMID17456828)  Entities that have same spelling but different meaning depending on the context, such as “code” in this sentence was not related to phenotype standard codes. | 4(8%) | 3(6%) | 7 (7%) |
| Clinical dictionary | “Uses inhaled steroids regularly.” (PMID12952547)  "inhaled steroids" was not recognized as the term not in our dictionary. | 25(50%) | 12(24%) | 37 (37%) |
| Keywords | “Baseline characteristics were compared using a X2 test for categorical variables and ANOVA for continuous variables.” (PMID15323063)  X2 test and ANOVA should be recognized as keywords for negative cues | 8(16%) | 27(54%) | 35 (35%) |
| Positive atypical | “Uses inhaled steroids regularly.” (PMID12952547)  This sentence is an actual positive and predicted as negative. These sentences with positive phenotyping context but no clear evidence. | 23(46%) | 0(0%) | 23 (23%) |
| Negative atypical | “Multivariate logistic regression identified patient factors associated with a correct diagnosis.” (PMID17712071)  This sentence is an actual negative and predicted as positive. It has many features for positive sentences. In addition, “regression” was recognized as a clinical diagnosis (phenotype). | 0(0%) | 40(80%) | 40 (40%) |

References

[1] Shatkay H, Craven M. Mining the biomedical literature: MIT Press; 2012.
